# Severe cytotoxic edema after CAR-T cell therapy for acute lymphoblastic leukemia

**DOI:** 10.1055/s-0046-1825766

**Published:** 2026-07-17

**Authors:** Victor Rebelo Procaci, Maria Luiza Lacerda, Luis Filipe de Souza Godoy, João Victor Luisi de Moura, Angela Sauter Dalbem, Mariana Nassif Kerbauy, Lucila Nassif Kerbauy

**Affiliations:** 1 Universidade Federal de São Paulo, Escola Paulista de Medicina, Disciplina de Neurologia, Setor de Neurologia Geral e Ataxias, São Paulo SP, Brazil.; 2 Hospital Israelita Albert Einstein, Departamento de Neurologia, São Paulo SP, Brazil.; 3 Hospital Israelita Albert Einstein, Departamento de Neurorradiologia, São Paulo SP, Brazil.; 4 Hospital Israelita Albert Einstein, Departamento de Hematologia, São Paulo SP, Brazil.

**Figure 1:**
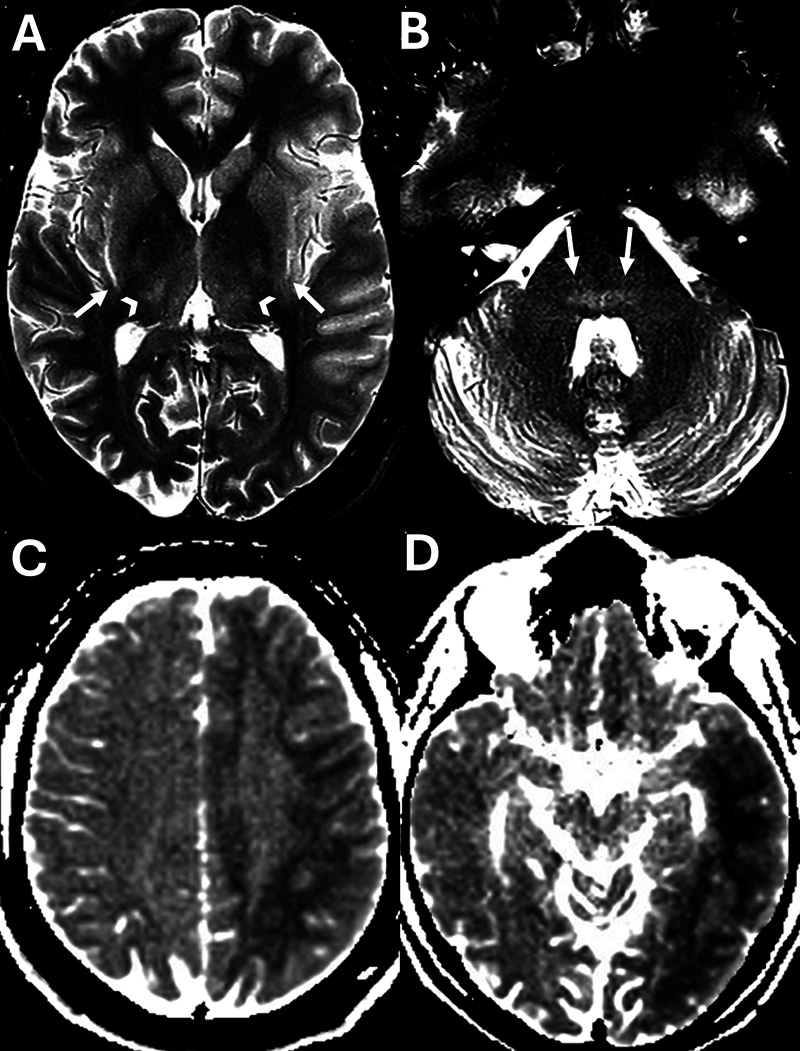
First T2 images, after immune effector cell-associated neurotoxicity syndrome (ICANS) symptoms developed. ( **A** , **B** ) Show hyperintensities in both thalami (arrowheads), external capsules, predominantly right-sided (arrows in **A** ), and pons tegmentum (arrows in **B** ). Apparent diffusion coefficient (ADC) maps ( **C** , **D** ) demonstrate extensive restricted diffusion, manifested as low signal intensity consistent with cytotoxic edema, predominantly affecting the subcortical white matter of the left hemisphere, with associated cortical involvement. The imaging pattern is not specific for ICANS and should be interpreted in conjunction with the appropriate clinical findings.

A 23-year-old man with relapsed acute lymphoblastic leukemia underwent CD19 chimeric antigen receptor modified T (CAR-T) cell therapy. He developed grade 1 cytokine release syndrome on day 1 and progressed to grade 4 immune effector cell-associated neurotoxicity syndrome (ICANS) by day 6, presenting with headache, somnolence, right hemiparesis, generalized seizures, and coma. Baseline magnetic resonance imaging (MRI) was normal. Follow-up scans ( Figures 1 2 ) on days 7, 16, and 163 showed severe hemispheric cytotoxic edema and multifocal T2/fluid-attenuated inversion recovery (FLAIR) hyperintensities, gradually resolving after tocilizumab, anakinra, and methylprednisolone. Acute deficits resolved completely, though the patient developed epilepsy, which was controlled with levetiracetam. Consistent with recent literature, 1 this case highlights the dynamic evolution of ICANS-related MRI abnormalities after CAR-T therapy.

**Figure 2:**
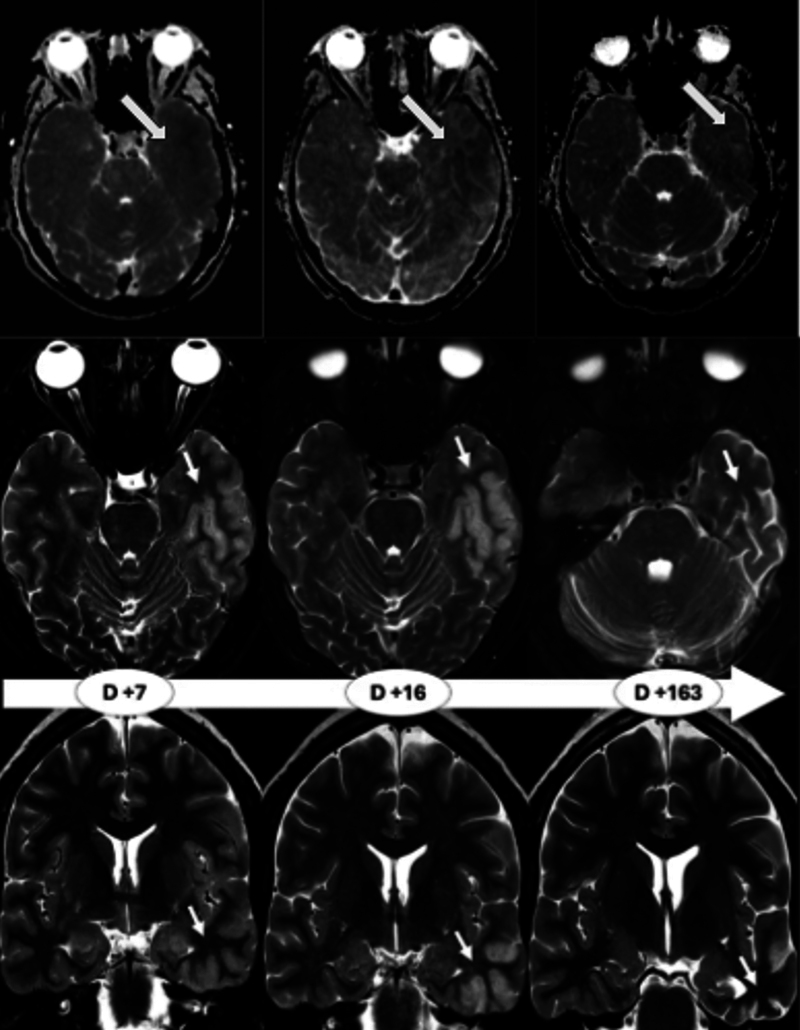
Follow-up magnetic resonance imaging (MRI) scans obtained at days 7, 16, and 163 demonstrates evolving cortical hyperintensities (arrows), which increase between days 7 and 16 and subsequently regress, with reexpansion of cortical sulci, parenchymal signal normalization, and residual temporal lobe atrophy. The middle and lower rows correspond to axial and coronal T2-weighted images, respectively. The upper row shows ADC maps demonstrating restricted diffusion, appearing as low signal intensity consistent with cytotoxic edema on days 7 and 16, with resolution of diffusion restriction by day 163, a pattern that may be observed following seizure activity.
